# Six Cell Cycle-related Genes Serve as Potential Prognostic Biomarkers and Correlated with Immune Infiltrates in Hepatocellular Carcinoma

**DOI:** 10.7150/jca.76809

**Published:** 2023-01-01

**Authors:** Ying Shi, Xiaopu Sang, Jiali Deng, Yihang Wang, Xiaoni Chen, Shan Lin, Fenfang Wu, Anlong Xu

**Affiliations:** 1 State Key Laboratory of Biocontrol, Guangdong Province Key Laboratory of Pharmaceutical Functional Genes, College of Life Sciences, Sun Yat-Sen University, Guangzhou, China.; 2 School of Life Sciences, Beijing University of Chinese Medicine, Beijing, China.; 3 Department of Central Laboratory, Shenzhen Hospital, Beijing University of Chinese Medicine, Shenzhen, China.

**Keywords:** cell cycle-related genes, hepatocellular carcinoma, tumor-infiltrating, prognostic biomarker, immune

## Abstract

**Background:** Cell cycle-related genes (*CDK1*, *CDK5*, *CDC20*, *CCNA2*, *CCNB1*, and *CCNB2*) play important roles in the regulation of mitotic cell cycle in eukaryotes. However, the correlation between cell cycle-related genes and tumor-infiltrating and prognosis of hepatocellular carcinoma (HCC) needs further investigation.

**Methods:** Two public websites, Tumor Immune Estimate Resource (TIMER) and Oncomine, were used to assess the expression levels of cycle-related genes. We also analyzed the protein expression levels of six cell cycle-related genes using the HPA database. In addition, Kaplan-Meier plotter and Gene Expression Profiling Interactive Analysis (GEPIA) database were used to investigate the impact of cell cycle-related gene expression levels on the clinical prognosis of HCC. The correlation between cell cycle-related genes and cancer immune infiltrates was analyzed through TIMER site. Subsequently, GEPIA and TIMER database were used to assess the correlation between the expression of six cell cycle-related genes and polygenic markers in monocytes and macrophages, respectively. The cell cycle-related genes were also analyzed to find the associated genes with the highest alteration frequency, by the Kyoto Encyclopedia of Genes and Genomes (KEGG) and Gene Ontology (GO) approaches of Metascape and String database, respectively.

**Results:** The expression levels of cell cycle-related genes were up-regulated in tumor tissues compared with normal tissues. Subsequently, the expression of high cell cycle-related genes was positively correlated with poor overall survival (OS) and progression-free survival (PFS) in HCC, for *CDK1* (OS: HR = 2.15, *P* = 1.1E-05 PFS: HR = 2.03, *P* = 2.3E-06),* CDK5* (OS: HR = 1.85, *P* = 0.0035 PFS: HR = 1.26, *P* = 0.17), *CDC20* (OS: HR = 2.49, *P* = 5.1E-07 PFS: HR = 1.77, *P* = 0.00012), *CCNA2* (OS: HR = 1.92, *P* = 0.00018 PFS: HR = 1.96, *P* = 5.2E-06), *CCNB1* (OS: HR = 2.34, *P* = 3.4E-05 PFS: HR = 1.97, *P* = 5.3E-06), and *CCNB2* (OS: HR = 1.91, *P* = 0.0013 PFS: HR = 1.63, *P* = 0.0011), respectively. Furthermore, the transcription level of cell cycle-related genes was significantly correlated with immune infiltrating levels of CD4+ T and CD8+ T cells, neutrophils, macrophages, and dendritic cells (DCs) in HCC, respectively. Amongst them, the expression levels of *CDK1*, *CDC20*, *CCNA2*, *CCNB1* and *CCNB2* manifest strongly correlated with diverse immune marker sets in HCC.

**Conclusions:** Our results demonstrated that cell cycle-related genes played key roles in the prognosis of HCC. Meanwhile, they were significantly correlated with immune infiltrating levels of CD4^+^ T cells, CD8^+^ T cells, neutrophils, macrophages and DCs in HCC, respectively. In addition, *CDK1*, *CDC20*, *CCNA2*, *CCNB1* and *CCNB2* expressions may be involved in the regulation of monocytes and tumor-associated macrophages (TAMs) in HCC, respectively. These findings strongly suggested that cell cycle-related genes could be used as novel biomarkers for exploring the prognosis and immune cells infiltration of HCC.

## Introduction

The hepatocellular carcinoma (HCC) is the third most prevalent cause of cancer-related deaths in worldwide, with more than 800,000 new cases annually all over the world 1, 2. In particular, HCC has the highest incidence in China due to susceptibility to hepatitis B virus (HBV) infection. Only 30% of HCC patients are diagnosed at an early stage and are amenable to reasonable treatment by tumor resection or liver transplantation 3. Although treatments (e.g., targeted therapeutics) and clinical medicine (e.g., sorafenib and regorafenib) have improved, but these therapies still do not improve satisfactory overall survival (OS) and progression-free survival (PFS) among clinical patients with HCC. Immune infiltration-related mechanisms play a key role in multiple cancer types, and immunotherapy may be considered as a promising approach for the treatment of HCC in future. Recently it has been reported that cytotoxic T lymphocyte antigen 4 (CTLA4) and programmed cell death 1 (PD1) complement each other functionally while protecting the body from pathogens and neoplasia 4, 5. Another immunotherapeutic method is the chimeric antigen receptor (CAR) T cell therapy that has been used to treat refractory disease, such as leukemia and lymphoma, successfully 6. In recent years, numerous studies has been reported that the multiple tumor-infiltrating lymphocytes, dendritic cells, monocyte, and tumor-associated macrophages (TAMs) affect the prognosis through greatly efficacy of chemotherapy and immunotherapy in HCC 7. Therefore, there is an urgent and meaningful need to find specific biomarkers to predict the prognosis and to guide the therapeutic goals of HCC. Recently, the public bioinformatics platform of molecular targets and networks provides a rich and convenient resource for cancer biomarker research, which can better explore the pathogenesis of HCC and identify novel functional genomic targets for therapeutic intervention.

Cell cycle-related genes play important roles in the regulation of the cycle by controlling the mitotic onset and centrosome cycle in eukaryotic cell; effectively regulating G1 progress and G1-S transition, and promoting G2-M transition 8. Researches showed that the disorder in cell cycle may lead to malignancy 9, 10. However, the underlying functions and regulatory mechanisms of cell cycle-related genes in HCC progression and tumor immunology are not fully elucidated.

Our study aimed to comprehensively explored cell cycle-related genes (cyclin dependent kinase 1: *CDK1*, cyclin dependent kinase 5: *CDK5*, Cell-division cycle protein 20 homologue: *CDC20*, Cyclin A2: *CCNA2*, Cyclin B1: *CCNB1*, and Cyclin B2: *CCNB2*) expression levels and correlation with prognosis features of HCC patients based on the online databases. These six-cell cycle-related genes not only control G2/M phase, but also function through the formation of specific serine/threonine protein kinase holoenzyme complexes with the cyclin-dependent protein kinases. Then, we analyzed the correlation of these genes with diverse tumor-infiltrating immune cells in HCC immune microenvironments via TIMER. This findings shed light on the key role of cell cycle-related genes in HCC, as well as provide an underlying molecular mechanism for tumor-immune interactions.

## Materials and methods

### Oncomine Database Analysis

Oncomine (https://www.oncomine.org) is a freely available web-based data-mining platform contain 65 gene expression datasets. The Oncomine database was used to analyze the transcription levels of the six-cell cycle-related genes in 20 cancers compared with normal tissues 11,12. The threshold level was measured as follow: *P*-value ≤ 0.01, fold change (FC) ± 2 and rank of top 10% genes.

### Gene Expression Profiling Interactive Analysis (GEPIA) Dataset

GEPIA is a convenient and versatile bioinformatics analysis platform that enables comprehensive analysis of material RNA sequencing through the TCGA and GTEx data with the normalized processing pipeline. The mRNA expression levels of cell cycle-related genes was analyzed by GEPIA in HCC dataset, and the profiling and pathologic stage, overall survival and correlation were analyzed with the following values of *P*-value ≤0.01, FC ± 2.

### The Human Protein Atlas Database Analysis

The public protein database, Human Protein Atlas (HPA, https://www.proteinatlas.org/), contains immunohistochemistry-based expression data for approximately 20 most common types of cancers. We used HPA database to analyze the protein expression levels of six cell cycle-related genes in normal tissues and HCC tissues, respectively.

### Cell culture

The HepG2 cells, BEL-7402 cells and LO2 cells were cultured in Dulbecco's Modified Eagle Medium (Gibco) containing 10% fetal bovine serum (Gibco), 100 μg/mL streptomycin sodium and 100 U/mL penicillin G sodium (MP Biomedicals) at 37 °C and 5% CO2.

### Real-time quantitative PCR

Total cell RNA was isolated using RNAiso (Takara). 500 ng of total RNA was used for reverse transcription with PrimeScrip RT reagent Kit (Takara). Real-time quantitative PCR was performed using SYBR Premix Ex Taq (Takara) with the following primer: CDK1 (Forward: AAAATTGGAGAAGGTACCTATGGA, Reverse: CCCTTCCTCTTCACTTTCTAGTCTG), CDK5 (Forward: CGCCGCGATGCAGAAATACGAGAA, Reverse: TGGCCCCAAAGAGGACATC), CDC20 (Forward: CTGGGTTCCTCTGCAGACAT, Reverse: CGAATGTGTCGATCACTGGT), CCNA2 (Forward: CGCTGGCGGTACTGAAGTC, Reverse: GAGGAACGGTGACATGCTCAT), CCNB1 (Forward: AATAAGGCGAAGATCAACATGGC, Reverse: TTTGTTACCAATGTCCCCAAGAG) and CCNB2 (Forward: GCGTTGGCATTATGGATCG, Reverse: TCTTCCGGGAAACTGGCTG).

All steps were performed as described in the user manual of the kit.

### Immunofluorescence

Cells were fixed using 4% paraformaldehyde for 15 min at room temperature and then washed 3 times with PBS. Cells were permeabilized by incubation in 0.2% Triton X-100 (Sigma-Aldrich) at room temperature for 15 min. Cells were blocked with 5% BSA, and incubated with CDK1 antibodies (Sangon Biotech, D190678), CDK5 antibodies (Sangon Biotech, D199878), CDC20 antibodies (Sangon Biotech, D120392), CCNA2 antibodies (Sangon Biotech, D160233), CCNB1 antibodies (Sangon Biotech, D260234), and CCNB2 antibodies (Sangon Biotech, D260235), at 4 °C overnight. Subsequently, cells were incubated with secondary antibodies in 1% donkey serum for 45 min at RT. Finally, the nuclei were counterstained with DAPI. Images were captured on an Olympus FV1000 Confocal Laser Scanning microscope. Leica TCS-SP5 confocal microscope. LAS X software (Leica) was used for image processing.

### Kaplan-Meier Plotter Database Analysis

Kaplan-Meier plotter (http://kmplot.com/analysis/index.php?p=service&cancer=liver_rnaseq) is an online platform for analyzing the correlation between the expression of the 54,675 genes on tumor survival rates in more than 20 different cancers through 10,461 cancer samples, including 364 liver cancer samples on the HGU133 Plus 2.0 array 13. A correlation analysis was conducted between *CDK1*, *CDK5*, *CDC20*, *CCNA2*, *CCNB1* and *CCNB2* mRNA transcription expression levels and survival rates in HCC using Kaplan-Meier survival curve. We also calculated the HR with 95% confidence intervals and log-rank *P*-value, with the following values of *P*-value ≤ 0.01, fold change ± 2.

### TIMER Database Analysis

The newly designed free web server, TIMER database (https://cistrome.shinyapps.io/timer/) 14 contains 10,897 samples from TCGA for comprehensive analysis of immune infiltrates information across 32 different cancer types. We analyzed the cell cycle-related gene expression in HCC and correlated the expression with the all kinds of immune infiltrates cells, such as B cells, CD8+ T cells, CD4+ T cells, macrophages, neutrophils and dendritic cells through gene modules 15, 16. Meanwhile, correlations between cell cycle-related genes mRNA expression levels and multiple gene markers of tumor-infiltrating immune cells were also estimated through correlation modules. The gene expression level was displayed with log_2_ RSEM.

### HCCDB Database Analysis

The HCCDB (http://lifeome.net/database/hccdb) is an available online database containing 15 public HCC expression datasets across 3917 clinical samples to exploring gene expression and clinic-pathological features of HCC. The expression level of cell cycle-related genes was confirmed by HCCDB database 17. The *P*-value was calculated by student's t-test. The *P* value and threshold fold change were set at 0.01 and 2.

### Metascape Database Analysis

The online bioinformatics analysis platform, Metascape (http://metascape.org), contains comprehensive bioinformatic knowledge bases, which is used to extract the enriched pathways and construct the protein-protein interaction (PPI) network based on the lists of multiple proteins and gene identifiers 17. The six-cell cycle-related genes were analyzed using the KEGG and GO methods of Metascape database, finally the linked genes with the highest alteration frequency were found.

### UALCAN Database Analysis

The UALCAN (http://ualcan.path.uab.edu/index.html) is a comprehensive online analysis tool; it enables the analysis of mRNA expression differences to compare normal samples with primary tumor tissue samples, as well as the access to different tumor pathological stages, detection of tumor grades and other clinic-pathologic features 18.

### STRING Database Analysis

The STRING (https://string-db.org/) is available online network. The STRING database is to create an exhaustive and convenient global platform, including PPI. For the enrichment analysis, STRING implements well-known classification systems and offers unique classification systems that rely on high-throughput data mining 19, 20.

### Statistical Analysis

The results analysis by Oncomine database are showed with *P* value, threshold fold changes, and ranks. The results obtained in Kaplan-Meier plots and GEPIA database are displayed with HR and *P* value from a log-rank test. The correlation of gene expression was generated by statistical significance: *P <0.05, **P <0.01, ***P <0.001.

## Results

### The Expression Levels of Cell Cycle-related Genes and Correlation with Pathological Parameters in HCC

**Figure 1:**
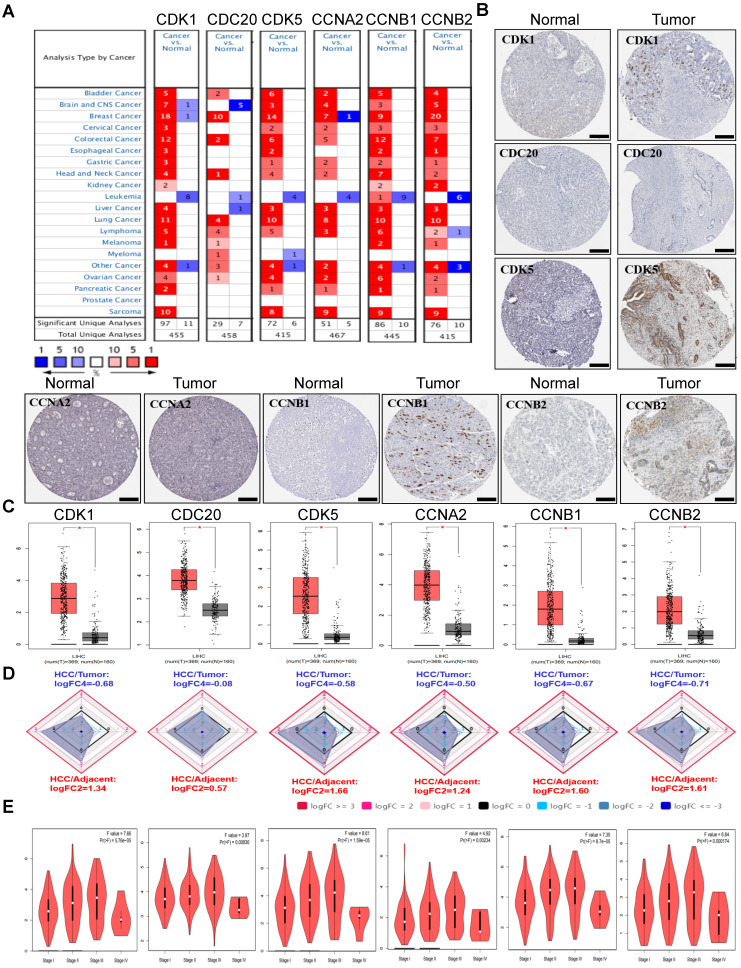
The expression levels of CDK1, CDK5, CDC20, CCNA2, CCNB1 and CCNB2 in HCC. **(A)** Transcriptional pattern of *CDK1*, *CDK5*, *CDC20*, *CCNA2*, *CCNB1* and *CCNB2* in major malignant tumors compared with those in normal tissues using the Oncomine database. The heat-map represents data with statistically significant upregulation (red) or downregulation (blue). The numbers in the heat-map indicate the published independent datasets of mRNA microarray experiments. **(B)** The protein expression levels of CDK1, CDK5, CDC20, CCNA2, CCNB1 and CCNB2 in HCC from HPA. Scale bar: 200μm. **(C)** The mRNA expression levels of *CDK1*, *CDK5*, *CDC20*, *CCNA2*, *CCNB1* and *CCNB2* in GEPIA. (*P < 0.05). **(D)** The mRNA expression levels of *CDK1*, *CDK5*, *CDC20*, *CCNA2*, *CCNB1* and *CCNB2* compared with the normal tissues in HCCDB. **(E)** Correlation of the *CDK1*, *CDK5*, *CDC20*, *CCNA2*, *CCNB1* and *CCNB2* expression levels with various tumor stages among HCC tissues in GEPIA.

To study the different expression levels of cell cycle-related genes in HCC patients, the *CDK1*, *CDK5*, *CDC20*, *CCNA2*, *CCNB1* and *CCNB*2 mRNA expression levels in tumor and normal sample from 20 types of malignant tumors were analyzed using the Oncomine database. Our findings demonstrated that mRNA expression levels of six cell cycle-related genes were all remarkably increased among patients with HCC **(Figure 1A)**. In addition, lower expression levels were observed in leukemia, melanoma and prostate cancer in some data sets. To further assesse cell cycle-related genes expression in HCC, we evaluated CDK1, CDK5, CDC20, CCNA2, CCNB1 and CCNB2 expression using the protein data in HPA database. It revealed from HPA database analysis that protein levels of CDK1, CDK5, CDC20, CCNA2, CCNB1 and CCNB2 were obviously upregulated in the HCC tissues compared with the normal tissues. Specifically, we compared the mRNA levels of six cell cycle-related genes of the tumor tissues with the adjacent normal tissues through the GEPIA, Ualcan and HCCDB database of HCC in TCGA (tumor sample: n = 369 vs. normal sample: n = 160) **(Figure 1B**-**D, Supplementary Figure 1)**. The result also showed that *CDK1*, *CDK5*, *CDC20*, *CCNA2*, *CCNB1* and *CCNB2* mRNA expression levels within tumor tissues were significantly increased compared with the adjacent normal tissues. The *P* values were listed in **Table 1** for this six-cell cycle-related genes from 12 datasets in HCCDB. The *CDK1*, *CDK5*, *CDC20*, *CCNA2*, *CCNB1* and *CCNB2* expression levels (tumor sample: n = 369 *vs.* normal sample: n = 160) were upregulated in HCC group compared with the non-carcinoma group (Analysis by GEPIA dataset). And then, the association between the expression levels of these genes and the HCC stages was also analyzed. The expression levels of *CDK1* (*P* = 5.76E-05), *CDK5* (*P* = 0.00836), *CDC20* (*P* = 1.59E-05), *CCNA2* (*P* = 0.00234), *CCNB1* (*P* = 8.7E-05) and *CCNB2* (*P* = 0.000174) were significantly increased in every major stage, respectively (**Figure 1E, Supplementary Figure 2**), (*P* <0.05).

**Figure 2:**
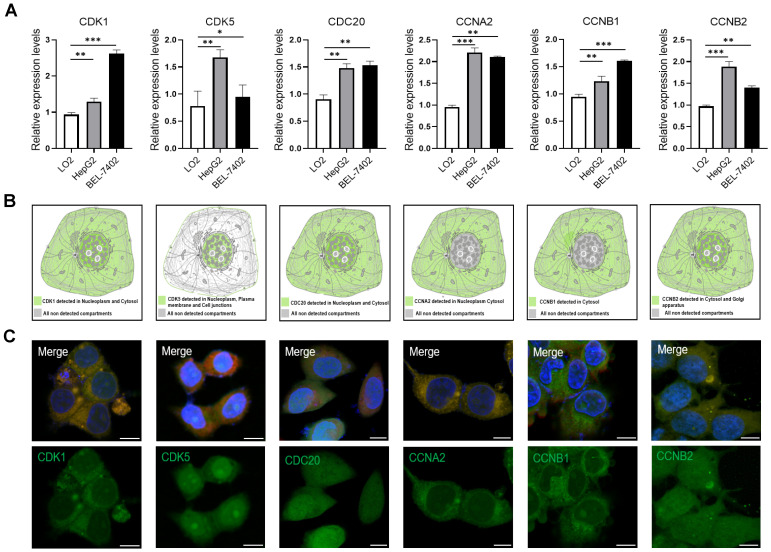
** (A)** The expression of *CDK1*, *CDK5*, *CDC20*, *CCNA2*, *CCNB1* and *CCNB2* in HepG2 and BEL-7402 cells compared with LO2 cells by quantitative PCR analysis. *p <0.05, **p <0.01, ***p <0.001; n = 3. **(B)** The subcellular localization of the six-cell cycle related protein analyzed in HPA database.** (C)** Immunofluorescence images of CDK1, CDK5, CDC20, CCNA2, CCNB1 and CCNB2 in HepG2 cells. The nucleuses were stained blue, the microtubules were stained red, and the cell cycle related proteins were stained green. Scale bar = 10μm.

To confirm our results from the database, we analyzed the expression levels of six cell cycle related genes in normal liver cells (LO2) and tumor liver cells (HepG2, BEL-7402) using quantitative PCR. The results showed that the expression levels of *CDK1*, *CDK5*, *CDC20*, *CCNA2*, *CCNB1* and *CCNB2* were significant up-regulated in HepG2 and BEL-7402 cells compare with LO2 cells **(Figure 2A)**. We also analyzed the subcellular localization of cell cycle-related proteins using the HPA database. In addition, immunofluorescence showed that CDK1, CDC20 and CCNB2 were mainly distributed in cytoplasm and nucleus **(Figure 2B, C)**. CCNA2 and CCNB1 were only distributed in cytoplasm, and CDK5 was only distributed in nucleus.

### Expression of Cell Cycle-related Genes in the Present of *TP53* Mutation Status in HCC

**Figure 3:**
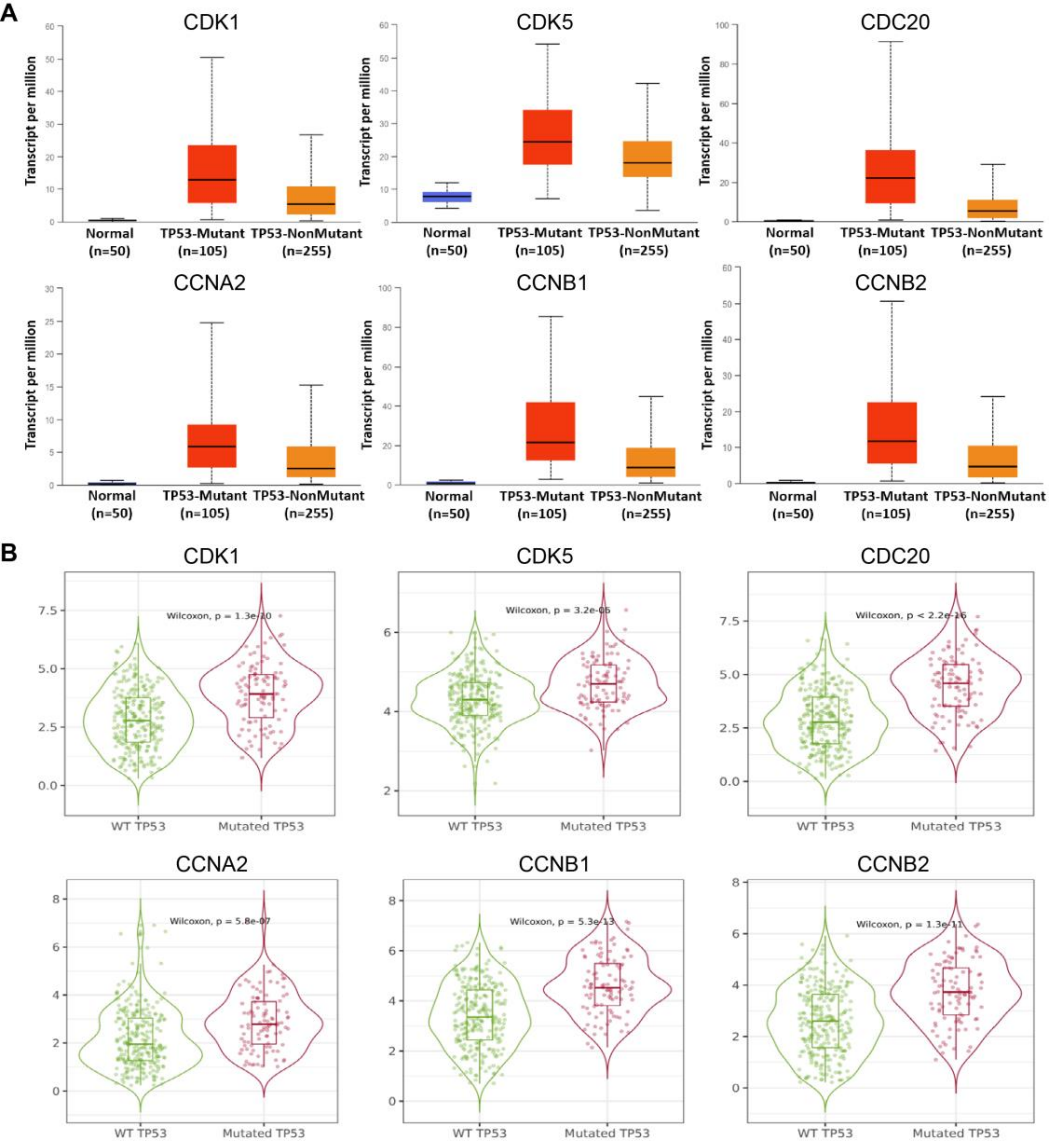
Expression levels of *CDK1*, *CDK5*, *CDC20*, *CCNA2*, *CCNB1* and *CCNB2* in HCC in the presence of *TP53* mutation status in UALCAN **(A)** and **(B)** GEPIA database.

As a tumor suppressor in cells,* TP53* lead to growth arrest and apoptosis depending on the physiological conditions and cell type 21.* TP53* mutation in HCC is associated with worse clinical stage and prognosis 22. Therefore, we individually analyzed the expression of 6 hub cell cycle-related genes in HCC when the *TP53* mutated. The results showed that the mRNA expression levels of *CDK1* (*P* = 8.44949999967426E-07), *CDK5* (*P* = 2.18779999999752E-05), *CDC20* (*P* = 3.42399997244058E-09), *CCNA2* (*P* = 2.377900E-02),* CCNB1* (*P* = 1.00539999658977E-08) and *CCNB2* (*P* = 1.34450000022213E-08) were significantly increased in* TP53*-mutant specimens compared to those without *TP53*-mutant (**Figure 3, Supplementary Table 1**). These results suggested that *TP53* mutation may an important role in the expression of cell cycle-related genes in HCC.

### Relationship between the Increased mRNA Expression of *CDK1*, *CDK5*, *CDC20*, *CCNA2*, *CCNB1* and *CCNB2* and Dismal Prognosis for HCC

**Figure 4:**
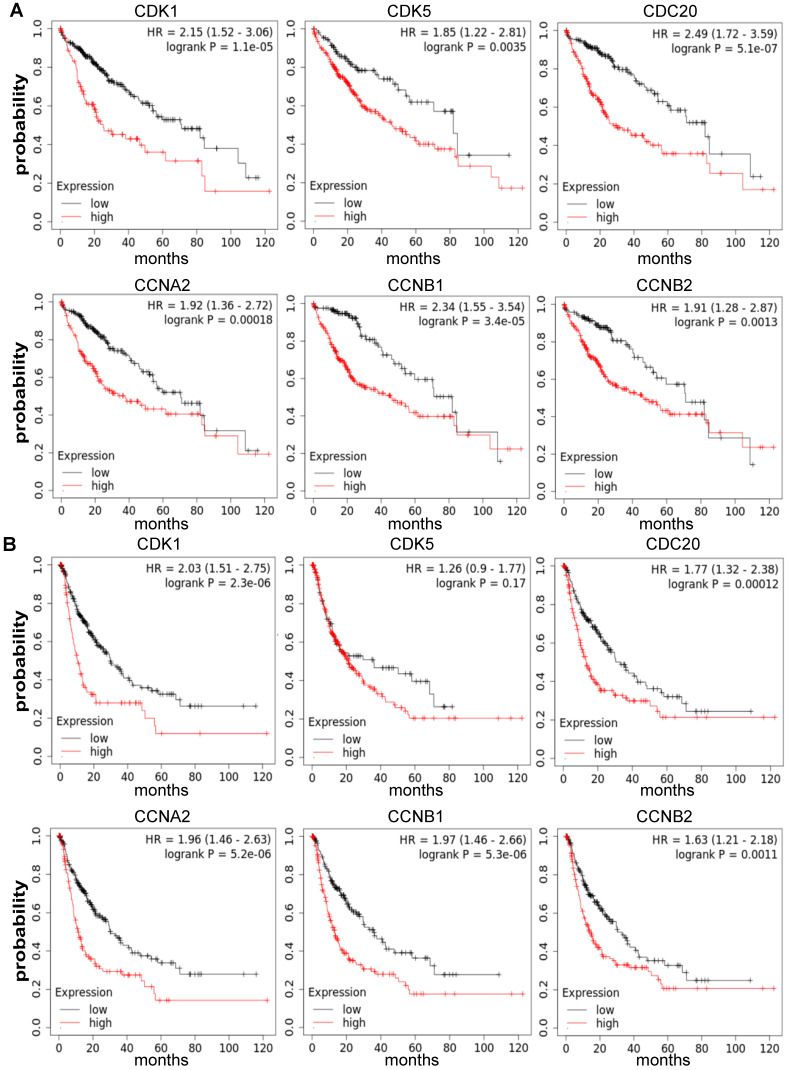
Contributions of *CDK1*, *CDK5*, *CDC20*, *CCNA2*, *CCNB1* and *CCNB2* mRNA expression levels in predicting prognosis of HCC patients (Kaplan-Meier plotter). **(A)** Relationship between high expression levels of* CDK1*, *CDK5*, *CDC20*, *CCNA2*, *CCNB1* and *CCNB2* with poor OS for HCC patients (n = 364). **(B)** Relationship between high expression levels of* CDK1*, *CDC20*, *CCNA2*, *CCNB1* and *CCNB2* with poor PFS for HCC patients (n = 370). OS, overall survival; PFS, Relapse free survival.

The crucial effect of cell cycle-related genes in HCC sample survival was also found. The Kaplan-Meier Plotter database was utilized to analyze the relationship between the *CDK1*, *CDK5*, *CDC20*, *CCNA2*, *CCNB1* and *CCNB2* mRNA expression and the outcome of HCC patients based on the public datasets. Our results displayed that the high expression levels of *CDK1* (OS: HR=2.15, *P* = 1.1E-05 PFS: HR = 2.03, *P* = 2.3E-06), *CDC20* (OS: HR = 2.49, *P* = 5.1E-07 PFS: HR = 1.77, *P* = 0.00012), *CCNA2* (OS: HR = 1.92, *P* = 0.00018 PFS: HR = 1.96, *P* = 5.2E-06), *CCNB1* (OS: HR = 2.34, *P* = 3.4E-05 PFS: HR = 1.97, *P* = 5.3E-06) and *CCNB2* (OS: HR = 1.91, *P* = 0.0013 PFS: HR = 1.63, *P* = 0.0011) were positively associated with poor OS and PFS (*P* <0.05). However, the increased expression level of *CDK5* (OS: HR = 1.85, *P* = 0.0035 PFS: HR = 1.26, *P* = 0.17) was significantly correlated with poor OS (*P* < 0.05), (**Figure 4, Supplementary Figure 3**). Therefore, high expression levels of cell cycle-related genes are considered to be an important risk factor for poor prognosis of HCC patients.

### High Expression Levels of Cell Cycle-related Genes Impact the Prognosis in HCC Patients with Risk Factors

Hepatitis virus and alcohol consumption are the main causes of HCC. Approximately 45% of cases could be attributed to hepatitis B virus (HBV) infection, 26% to hepatitis C virus (HCV) infection, and 20% to alcoholic liver disease 23. Then, Kaplan-Meier Plotter database was used to evaluate the correlation between the expression level of cell cycle-related genes and OS and PFS under different risk factors. *CDK1* (OS: *P* = 0.00091 PFS: *P* = 1.7E-05), *CDK5* (OS: *P* = 0.0093 PFS: *P* = 0.043), *CDC20* (OS: *P* = 0.0076 PFS: *P* = 7.8E-05), *CCNA2* (OS: *P* = 0.0069 PFS: *P* = 1.4E-07), CCNB1 (OS: *P* = 0.00064 PFS: *P* = 2.4E-07) and CCNB2 (OS: *P* = 0.0015 PFS: *P* = 0.00013) were up-regulated in HCC patients under poor OS and PFS with alcohol consumption. *CDK5* (OS: *P* = 0.0029), *CDC20* (OS: *P* = 0.0013), *CCNA2* (OS: *P* = 0.027), and CCNB1 (OS: *P* = 0.0019) were up-regulated in HCC patients under poor OS with hepatitis virus. *CDK1* (OS: *P* = 4E-04 PFS: *P* = 0.017) was up-regulated in those under poor OS and PFS with hepatitis virus.

### *CDK1*, *CDK5*, *CDC20*, *CCNA2*, *CCNB1*, and *CCNB2* Expression Is Correlated with Immune Infiltration Level in HCC

Tumor-infiltrating lymphocytes are considered to be an independent predictor of sentinel lymph node status in multiple tumors and survival 24. The free online database TIMER was used to analyze whether the expressions of *CDK1*, *CDK5*, *CDC20*, *CCNA2*, *CCNB1* and *CCNB2* were correlated with the level of HCC immune infiltration. Our results showed that the expression levels of cell cycle-related genes were significantly correlated with B cell, CD8+ T cells, CD4+ T cells, neutrophils, macrophages, and DCs in HCC **(Figure 6)**. The *P*-values were listed in **Supplementary Table 2**.

### Correlation Analysis between Expression of *CDK1*, *CDC20*, *CCNA2*, *CCNB1* and *CCNB2* and Marker Sets of Immune Cells

#### Correlation Analysis between Expression of CDK1, CDC20, CCNA2, CCNB1 and CCNB2 and Marker Sets of Immune Cells

In order to better analyze the relationship between expression of CDK1, CDC20, CCNA2, CCNB1 and CCNB2 and multiple immune infiltrates, we further investigated the relationship between the expression of these genes and the levels of multiple marker sets of different HCC immune cells in GEPIA and TIMER databases, respectively. Including monocytes (CFS1R, CD86), TAMs (CD68, CCL2, IL10), M1 macrophages (COX2, IRF5, INOS) and M2 macrophages (MS4A4A, VSIG4, CD163) in HCC (Table 2 and Figures 7).

Surprisingly, our results showed a strong correlation between the transcription levels of majority marker sets of monocytes, TAMs, and other immune cells and the expression levels of CDK1, CDK5, CDC20, CCNA2, CCNB1 and CCNB2 in HCC (Table 2, Supplementary Table 2). Furthermore, the expression levels of chemokine (C-C motif) ligand (CCL)-2, IL10 and CD68 of TAMs were significantly correlated with the expression levels of CDK1, CDC20, CCNA2, CCNB1 and CCNB2 in HCC (Figures 7A-B).

### Prediction of the Functions and Pathway Enrichment Analyses of* CDK1*, *CDK5*, *CDC20*, *CCNA2*, *CCNB1* and *CCNB2* among HCC Cases

The co-expression genes of *CDK1*, *CDK5*, *CDC20*, *CCNA2*, *CCNB1*, *CCNB2*,* BUB1*, *AURKA*, *PLK1*, *BUB1B*, *BUB3*, *MAD2L1*, *CDC6*, *NDC80*,* TP53* and *CDKN18* were analyzed using STRING database and functional protein association networks **(Figure 8A)**. Subsequently, the lists of all expressed cell cycle-related genes with the highest alteration frequency were compiled and analyzed using KEGG and GO methods from the Metascape database (**Figure 8B, C, D**). The results showed that changes in cell cycle-related genes affected the following cellular processes and pathways: cell cycle (hsa04110); positive regulation of cell cycle (GO: 0045787); regulation of cyclin-dependent protein serine/ threonine kinase activity (GO: 0000079); microtubule cytoskeleton organization (GO: 0000226), PLK1 PATHWAY (M129: PID).

## Discussion

Cell cycle transitions are pivotal events in regulating cell proliferation in eukaryotic cells. This complex and thorough process are regulated by the cyclin dependent kinases family (CDKs), which are actuated by interacting with specific cyclins 8, 25. This complex and thorough process is regulated by a family of cyclin dependent kinases family (CDKs), which are actuated by interacting with specific cyclins 8, 25. CDK1 and CDK5, as main members of the CDK family, are originally known as threonine/serine-specific protein kinases activated to initiate the cell cycle. CDK1 has been found to be active in several tumor-regulating cell adhesion cycles and can be identified as a prognostic biomarker for various types of cancer 26, 27. CDK5 is one of the most special members of the CDK family, which is one of the first CDK members to be found in non-cycling cells. There is increasing evidence that it can promote tumorigenesis in certain cellular environments 28. Cyclin B cluster, a member of the cyclin B1 and cyclin B2 families in human beings, is an important cell cycle control protein and effectively regulates the cell entry into mitosis 29. Cyclins A2 regulates the cell cycle by promoting the entry and progression of the S phase 29. CDC20 is also an essential cell-cycle regulator, completing mitosis and mediating cell cycle-related protein-protein interactions 30, 31. However, few reports have described the prognostic characteristics of different cell cycle-related genes. Furthermore, there are few reports on the correlation between cycle-related genes and immune infiltration in HCC. Therefore, our current work provides a new perspective for exploring the potential function of cell cycle-related genes in tumor immunology, which can be regarded as biomarker for HCC.

Here we analyzed the expression levels and systemic prognostic status of 6 cell cycle-related genes in HCC using public independent dataset of Oncomine, HPA, GEPIA, and HCCDB. The results showed that the expression of CDK1, CDK5, CDC20, CCNA2, CCNB1 and CCNB2 in HCC tissues were higher than those in normal or adjacent tissues (**Figure 1**). Overexpression of *CDC20* and* CDK1* has been reported in various malignancies, which are also associated with high tumor grade in cervical, colon, and renal carcinomas 32-34. TCGA database also showed that increased transcription of cell cycle-related genes was correlated with poor HCC prognosis (**Supplementary Figure 1**). Next, these six-cell cycle-related genes with different major stages and grades of HCC were analyzed by GEPIA, TISIDB and Ualcan online database, respectively. The results showed that the transcription level of *CDK1*, *CDC20*, *CCNA2*, *CCNB1* and *CCNB2* in stage II and stage III was significantly increased than that in stage I in HCC (**Figure 1E, Supplementary Figure 2**). The results also showed that the expression level of *CDK1*, *CDC20*, *CCNA2*, *CCNB1* and *CCNB2* in grade II, grade III and grade Ⅳ were up-regulated compared to that in grade I. These results indicated that these cell cycle-related genes may play an important role in the end-stage of HCC. To confirm our results from the database, we analyzed the expression levels of six cell cycle related genes in normal liver cells (LO2) and tumor liver cells (HepG2, BEL-7402) using quantitative PCR and immunofluorescence. The results showed that the expression levels of six cell cycle related genes were significant up-regulated in HepG2 and BEL-7402 cells compare with LO2 (**Figure 2**). *TP53* mutations are considered to be the leading cause of HCC. Our results showed that the expression levels of *CDK1*, *CDK5*, *CDC20*, *CCNA2*, *CCNB1* and *CCNB2* were higher in *TP53*-mutant sample than that in *TP53*-nonmutant sample **(Figure 3, Supplementary Table 1)**. In addition, Kaplan-Meier Plotter database results showed that high cell cycle-related genes expression was associated with a low positive hazard ratio of OS and PFS in HCC patients **(Figure 4)**. These findings strongly suggest that cell cycle-related genes may be prognostic biomarker for HCC.

**Figure 5:**
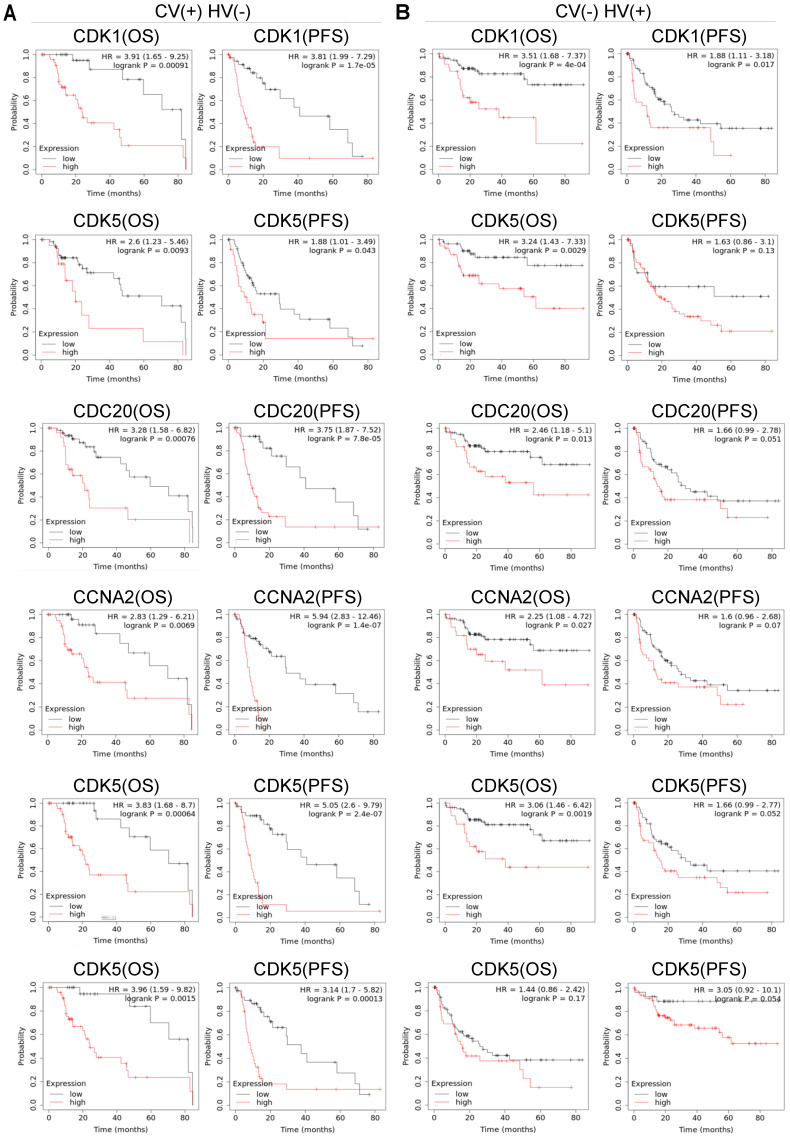
The relationship between the expression levels of *CDK1*, *CDK5*, *CDC20*, *CCNA2*, *CCNB1* and *CCNB2* and the prognosis of HCC with risk factors. **(A)** Relationship of up-regulation expression levels of six cell cycle-related genes with OS (n = 117) and PFS (n = 169) of HCC patients with AC, **(B)** The correlation between high transcription levels of six cell cycle-related genes with OS (n = 153) and PFS (n = 205) of HCC patients with HV.

Hepatitis B virus (HBV) and hepatitis C virus (HCV), along with alcohol abuse and metabolic syndrome are the most attributable causes in HCC 35. We also evaluated the association of cell cycle-related genes between OS and PFS under different risk factors at Kaplan-Meier Plotter database. The high expression of *CDK1*, *CDK5*, *CDC20*, *CCNA2*, *CCNB1* and *CCNB2* was associated with poor PFS and OS induced by alcohol consumption **(Figure 5)**. The high expression of *CDK5*, *CDC20*, *CCNA2* and *CCNB1* was only associated with poor OS of hepatitis virus. *CDK1* was up-regulated in dismal PFS and OS with hepatitis virus. However, the mechanistic relationship between six cell cycle-related genes and viral-infected HCC remains to be further explored.

**Figure 6:**
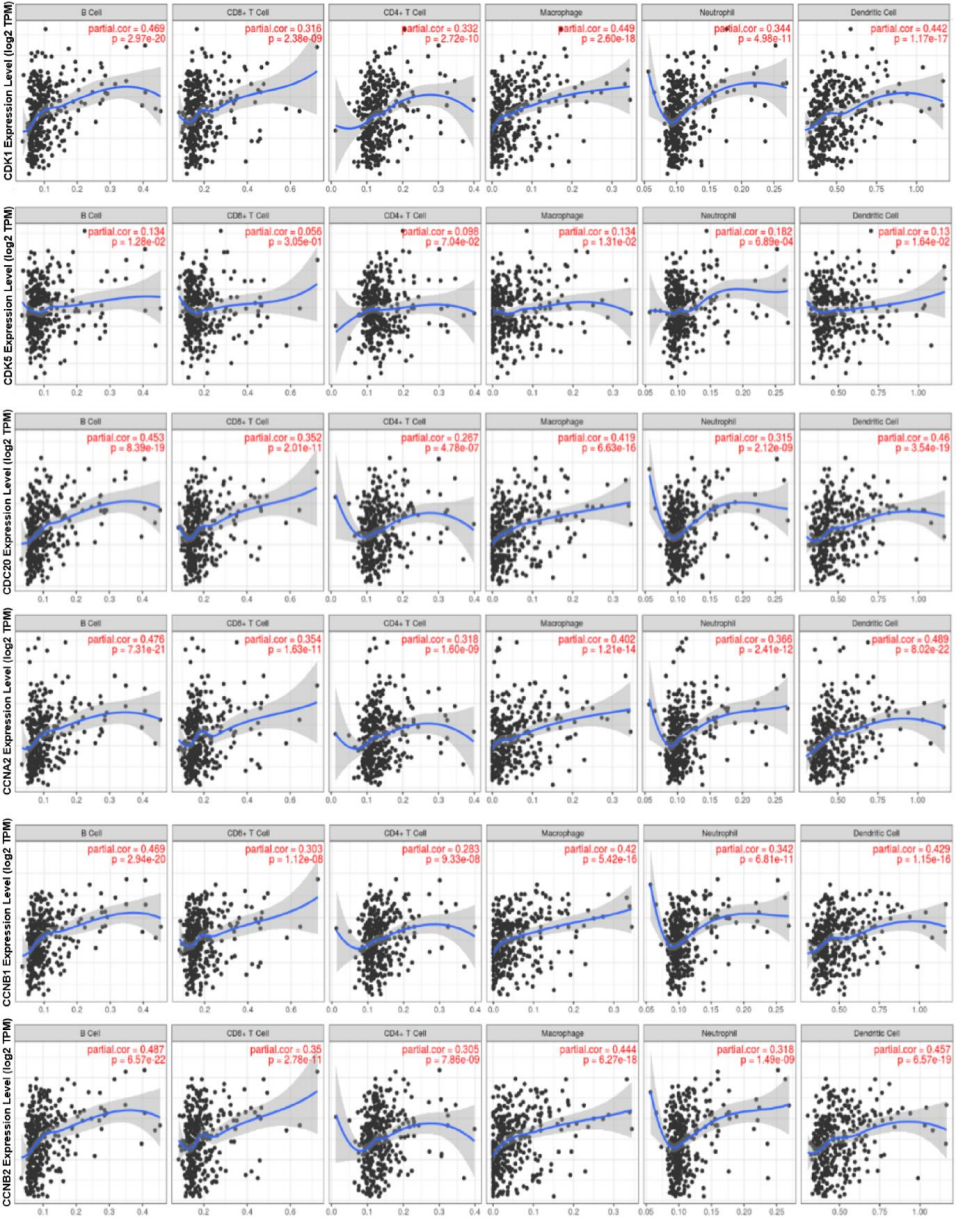
The relationship between the expression of *CDK1*, *CDK5*, *CDC20*, *CCNA2*, *CCNB1*, *CCNB2* and the level of HCC immune infiltration. The expressions of *CDK1*, *CDC20*, *CCNA2*, *CCNB1* and *CCNB2* were significantly positively correlated with the infiltration levels of B cells, CD8+ T cells, CD4+ T cells, neutrophils, macrophages and DCs in HCC (n = 457). *CDK5* was positively correlated with infiltrating level of B cell, Macrophage, neutrophils and DCs.

**Figure 7:**
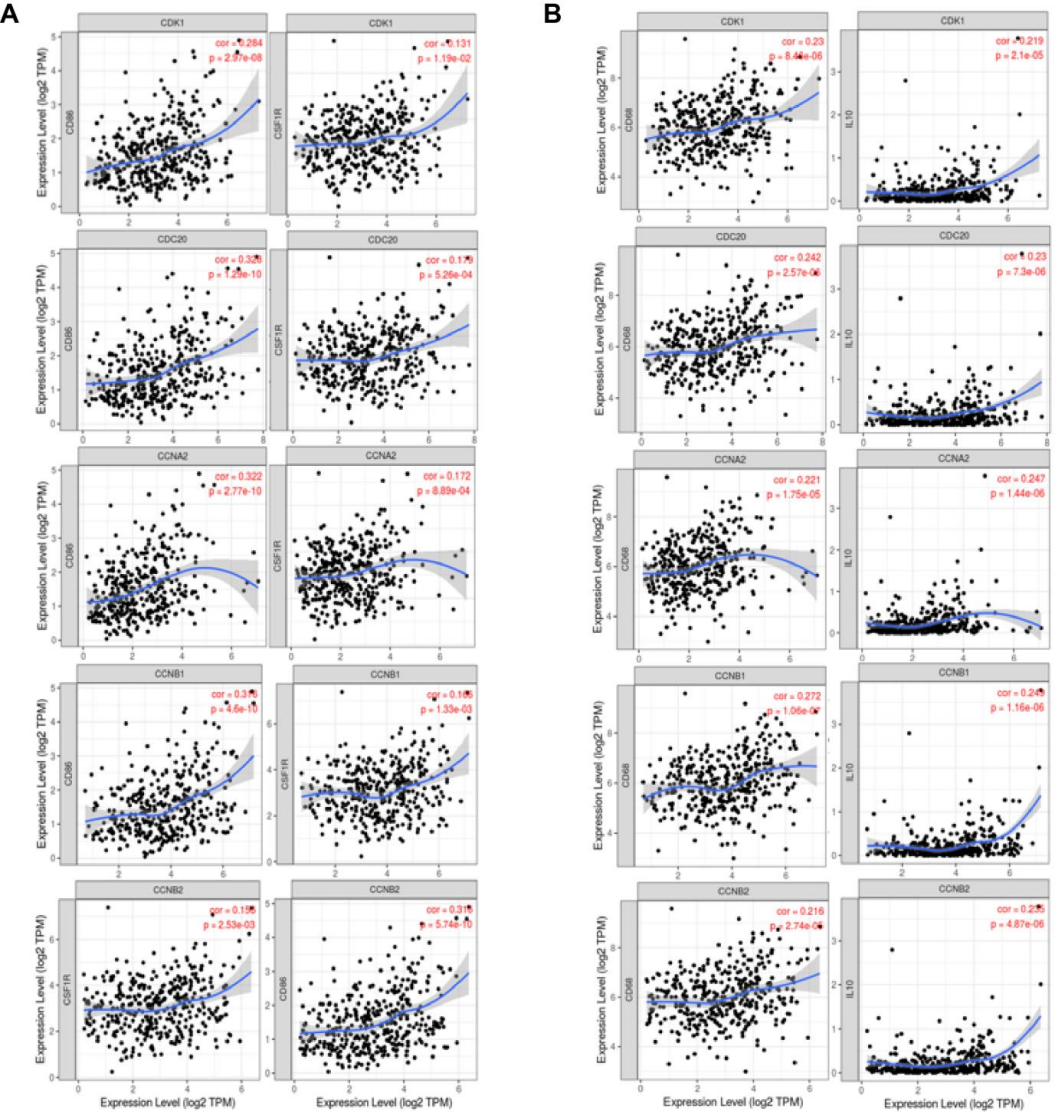
The expression levels of* CDK1*, *CDC20*, *CCNA2*, *CCNB1* and *CCNB2* were correlated with the polarization of macrophages in HCC. Markers included CSF1R and CD86 of monocytes; IL10 and CD68 of TAMs (tumor-associated macrophages). (**A**) Scatterplots of correlations between six cell cycle-related genes expression and gene markers of monocytes. (**B**) Scatterplots of correlations between six cell cycle-related genes expression and gene markers of TAMs.

Another important finding of our study was that cell cycle-related gene transcription was positively correlated with the infiltration level of B cell, CD8+ T cells, CD4+ T cells, macrophages, neutrophils and dendritic cells in HCC (**Figure 6, Supplementary Table 2**). Afterwards, the correlation between cell cycle-related gene expression and multiple immune cell marker genes suggests the role of these genes in regulating tumor immunology in HCC (**Figure 7**). Firstly, M1 macrophage markers such as PTGS2 were weakly correlated with cell cycle-related gene expression, while M2 macrophage markers such as MS4A4A, VSIG4 and CD163 were moderately or strongly correlated with *CCNA2* and *CCNB1* (**Table 2, Supplementary Table 3**). These results revealed the potential regulatory role of cell cycle-related genes in polarization of tumor-associated macrophages (TAM). In addition, TAMs-related immune interactions could be a potential indicator of cell cycle-related genes markers. Together our findings suggested that cell cycle-related genes play an important role in the recruitment and regulation of immune infiltrating cells in HCC.

**Figure 8:**
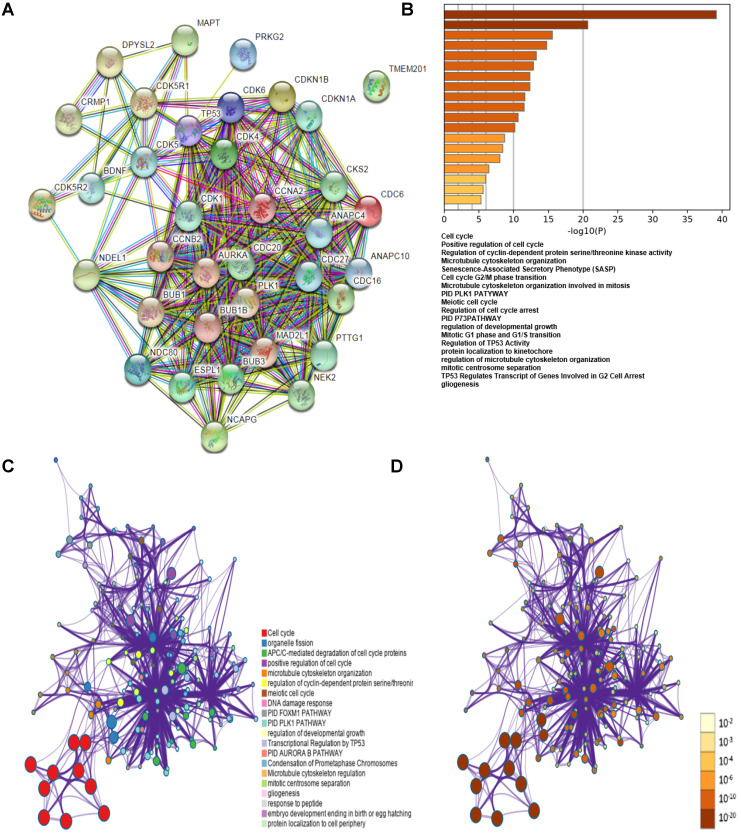
**Gene co-expression among HCC cases. (A)** Gene co-expression among HCC cases (STRING). **(B)** Functions of *CDK1*, *CDK5*, *CDC20*, *CCNA2*, *CCNB1* and *CCNB2* showed positive correlation with these genes alterations. **(C)** Network of KEGG and GO enriched terms colored by clusters. **(D)** Network of KEGG and GO enriched terms colored by *P*-value (Metascape database).

To further explore the mechanisms of cell cycle-related gene transcription associated with immune infiltration and poor prognosis, we constructed and analyzed related gene network using GO and KEGG methods (**Figure 8**). PLK1 (Polo-like kinase 1) plays a key role in regulating chromosome segregation, harmonizing centrosome, and mediating cytokinesis and meiosis 36-38. The PLK1 pathway has been revealed to play an indispensable role in patients with advanced solid malignancies, and its overexpression is associated with poor prognosis in patients with cancers, such as HCC 39, 40. Cell cycle-related genes may be involved in the PLK1 pathway to accelerate tumorigenesis.

In conclusion, we found that up-regulated expression of CDK1, CDK5, CDC20, CCNA2, CCNB1 and CCNB2 in HCC tissues was associated with poor prognosis, and significantly improved immune infiltration levels of CD8+T cells, CD4+ T cells, neutrophils, macrophages and DCs. Meanwhile, the high expression of these genes has a remarkably impact on the poor OS and PFS in HCC patients under viral infection or alcohol intake. Thus, we speculated that *CDK1*, *CDK5*, *CDC20*, *CCNA2*, *CCNB1* and *CCNB2* might be novel prognostic biomarkers and promising immunity therapeutic targets for HCC patients.

## Supplementary Material

Supplementary figures and tables.

## Figures and Tables

**Table 1:** *P*-value of the six up-regulated cell cycle-related genes in HCCDB database

Dataset	CDK1	CDK5	CDC20	CCNA2	CCNB1	CCNB2
HCCDB1	**5.010e-42**	**1.250e-22**	**6.990e-40**	**8.920e-50**	**6.970e-52**	**1.970e-55**
HCCDB3	**5.750e-53**	**1.740e-46**	**3.760e-36**	**5.030e-11**	**4.530e-47**	**7.410e-45**
HCCDB4	**2.460e-39**	**1.530e-55**	**1.870e-90**	**8.970e-59**	**8.720e-48**	**2.050e-82**
HCCDB6	**1.040e-67**	**8.120e-27**	**8.420e-64**	**1.590e-48**	**2.820e-87**	**3.200e-60**
HCCDB7	**5.190e-8**	0.8876	**3.800e-9**	**3.320e-7**	**6.830e-12**	**2.120e-7**
HCCDB11	0.5330	0.5333	**0.0007623**	0.2078	**0.00001180**	0.4481
HCCDB12	**3.540e-15**	**0.00002120**	**1.360e-10**	**8.250e-12**	**3.510e-16**	**6.790e-14**
HCCDB13	**1.510e-43**	**2.990e-41**	**3.690e-8**	**2.570e-43**	**2.310e-55**	**4.490e-47**
HCCDB15	**7.150e-28**	**9.000e-25**	**4.110e-29**	**2.690e-27**	**1.290e-38**	**1.810e-27**
HCCDB16	**7.480e-11**	**1.180e-13**	**9.120e-12**	**2.650e-14**	**1.710e-11**	**8.010e-15**
HCCDB17	**7.700e-17**	**1.180e-13**	**7.770e-14**	**1.170e-14**	**1.930e-17**	**8.420e-18**
HCCDB18	**3.710e-76**	**6.680e-71**	**2.630e-63**	**1.200e-57**	**1.200e-76**	**4.420e-63**

Bold values indicate statistically significant (*P* < 0.05).

**Table 2:** Correlation Analysis between Expression of *CDK1*, *CDK5*, *CDC20*, *CCNA2*, *CCNB1* and *CCNB2* and Levels of Markers of Immune Cells in TIMER

Description	Gene markers						
		**CDK1**		**CDK5**		**CDC20**	
		**Cor**	***P***	**Cor**	***P***	**Cor**	***P***
Monocyte	CD86	0.284	***	0.069	***	0.326	***
	CD115 (CSF1R)	0.131	*	0.088	0.0918	0.179	***
TAM	CCL2	0.039	0.459	0.02	0.701	0.038	0.464
	CD68	0.23	***	0.076	0.142	0.242	***
	IL10	0.219	***	0.012	0.822	0.23	***
M1 Macrophage	INOS (NOS2)	0.02	0.695	0.116	*	0.098	0.0588
	IRF5	0.394	***	0.367	***	0.325	***
	COX2(PTGS2)	0.101	0.0526	-0.109	*	0.034	0.514
M2 Macrophage	CD163	0.067	0.0197	0.043	0.406	0.046	0.38
	VSIG4	0.08	0.123	0.149	*	0.098	0.0588
	MS4A4A	0.089	0.0859	0.056	0.285	0.089	0.0869
		**CCNA2**		**CCNB1**		**CCNB2**	
		**Cor**	***P***	**Cor**	***P***	**Cor**	***P***
Monocyte	CD86	0.322	***	0.316	***	0.318	***
	CD115 (CSF1R)	0.172	***	0.166	**	0.156	*
TAM	CCL2	0.076	0.144	0.034	0.512	0.065	0.211
	CD68	0.221	***	0.272	***	0.216	***
	IL10	0.247	***	0.249	***	0.235	***
M1 Macrophage	INOS (NOS2)	0.022	0.674	0.028	0.597	0.014	0.786
	IRF5	0.369	***	0.377	***	0.379	***
	COX2(PTGS2)	0.125	*	0.08	0.124	0.078	0.133
M2 Macrophage	CD163	0.125	**	0.082	0.114	0.059	0.253
	VSIG4	0.125	*	0.106	**	0.077	0.141
	MS4A4A	0.133	*	0.106	*	0.087	0.0945

One asterisk: *P* < 0.05, two asterisks: *P* < 0.01, three asterisks: *P* < 0.001.
