# Supplementary material for: Six Cell Cycle-related Genes Serve as Potential Prognostic Biomarkers and Correlated with Immune Infiltrates in Hepatocellular Carcinoma
Source: J Cancer. 2023 Jan 1;14(1):9–23. doi: 10.7150/jca.76809 (PMC9809328; doi:10.7150/jca.76809)
Supplement: Supplementary file 1 — Supplementary figures and tables. [file jcav14p0009s1.pdf]

Six Cell Cycle-related Genes Are Prognostic Biomarkers and Correlated with Immune Infiltrates in Hepatocellular Carcinoma

Ying Shi[1], Xiaopu Sang[2], Jiali Deng[1], Xiaoni Chen[3], Fenfang Wu[1,3]*, Anlong Xu[1,2]*

[1]State Key Laboratory of Biocontrol, Guangdong Province Key Laboratory of Pharmaceutical Functional Genes, College of Life Sciences, Sun Yat-Sen University, Guangzhou, China; [2]School of Life Sciences, Beijing University of Chinese Medicine, Beijing, China; [3]Department of Central Laboratory, Shenzhen Hospital, Beijing University of Chinese Medicine, Shenzhen, China.

*Correspondence: Anlong Xu (xuanlong@bucm.edu.cn); Fenfang Wu (wufenfang19@126.com)

The Expression Levels of Cell Cycle-related Genes and Correlation with Pathological Parameters in HCC

We also examined cell cycle-related genes expression between the tumor and normal tissues using UALCAN database (tumor sample: n = 371 vs. normal sample: n = 50) (**Supplementary Figure 1**). The result showed that *CDK1* (*P* = 1E-12), *CDK5* (*P* = 1.62458935193399E-12), *CDC20* (*P* = 1.62436730732907E-12), *CCNA2* (*P* = 1.62447832963153E-12), *CCNB1* (*P* = 1E-12), and *CCNB2* (*P* = 1E-12) expression was significantly increased compared with normal tissues.

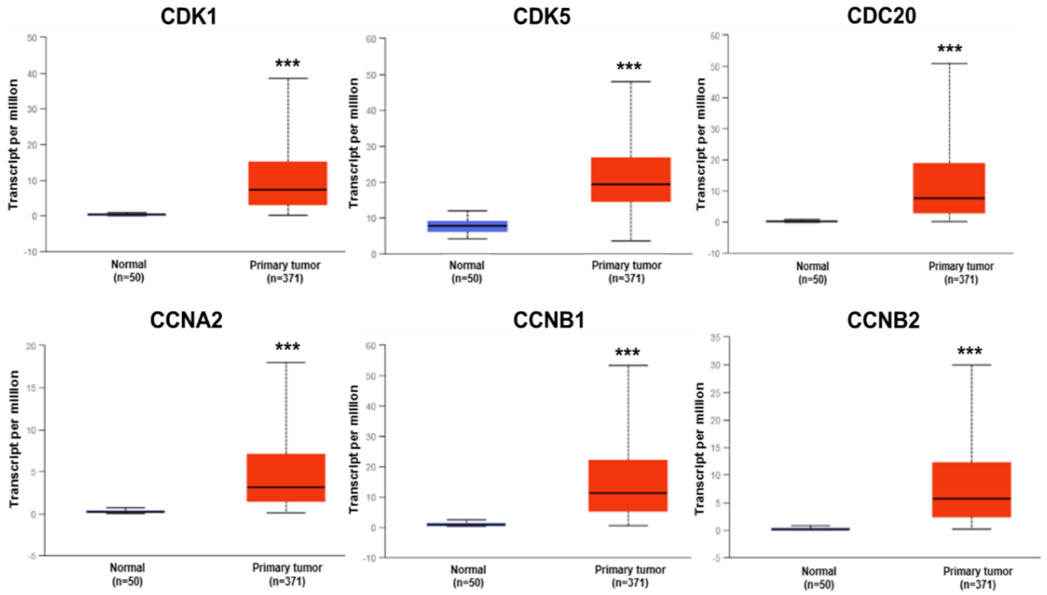

SUPPLEMENTARY FIGURE 1 | *CDK1*, *CDK5*, *CDC20*, *CCNA2*, *CCNB1*, and *CCNB2* expression increased in HCC presented in the form of a boxplot (Ualcan database). (****P* <0.001).

Expression of Cell Cycle-related Genes in HCC Based on *TP53* Mutation Status in UALCAN Database

We individually analyzed the expression of six hub cell cycle-related genes in HCC based on *TP53* mutation status. The results showed that the mRNA expression levels of *CDK1* (*P* = 8.44949999967426E-07), *CDK5* (*P* = 2.18779999999752E-05), *CDC20* (*P* = 3.42399997244058E-09), *CCNA2* (*P* = 2.377900E-02), *CCNB1* (*P* = 1.00539999658977E-08) and *CCNB2* (*P* = 1.34450000022213E-08) was significantly increased in *TP53*-mutant sample compared to *TP53*-

nonmutant (**Supplementary Table 1**).

SUPPLEMENTARY TABLE 1 | Expression of Cell Cycle-related Genes in HCC Based on *TP53* Mutation Status in UALCAN Database.

Gene name	TPM (median)		*P* value
	TP53-Mutant (105)	*TP53*-NonMutant (255)	
CDK1	12.931	5.496	**8.44949999967426E-07**
CDK5	24.333	18.114	**2.18779999999752E-05**
CDC20	22.126	5.238	**3.42399997244058E-09**
CCNA2	5.838	2.513	**2.377900E-02**
CCNB1	21.55	8.566	**1.00539999658977E-08**
CCNB2	11.719	4.731	**1.34450000022213E-08**

TPM, Transcript per million. Bold values indicate statistically significant (*P* <0.05).

Associations of *CDK1*, *CDK5*, *CDC20*, *CCNA2*, *CCNB1*, and *CCNB2* mRNA Expression with Clinico-pathological Variables in HCC Patients.

Additionally, the association of the expression of these genes with the HCC stage and grade was analyzed. UALCAN database showed that *CDK1*, *CDK5*, *CDC20*, *CCNA2*, *CCNB1* and *CCNB2* expression were significant differences in different stage and grade, respectively. (**Supplementary Figure 2**). Meanwhile, TISIDB database showed that *CDK1* (*P* = 7.26E-05), *CDC20* (*P* = 0.000101), *CCNA2* (*P* = 0.000332), *CCNB1* (*P* = 5.01E-05) and *CCNB2* (*P* = 0.000557) high expression in stage 2 and 3 compared with normal and stage 1. The results also showed that *CDK1* (*P* = 1E-10), *CDC20* (*P* = 1.3E-10), *CCNA2* (*P* = 2.28E-7), *CCNB1* (*P* = 9.67E-12) and *CCNB2* (*P* = 3.79E-10) high expression in grade 2, 3 and 4 compared with normal and stage 1.

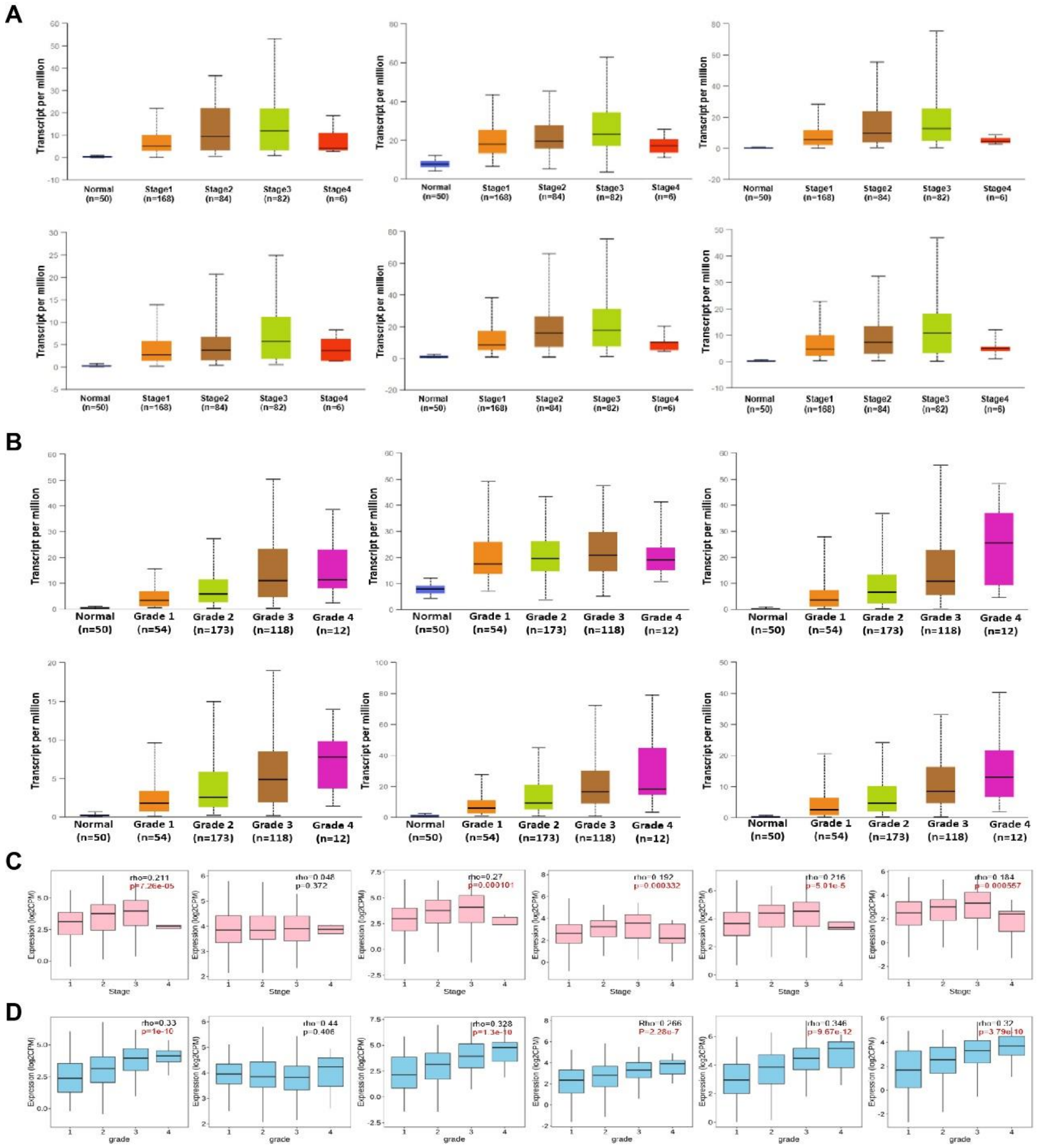

SUPPLEMENTARY FIGURE 2 | A. Correlation of the *CDK1*, *CDK5*, *CDC20*, *CCNA2*, *CCNB1*, and *CCNB2* expression with tumor stage among HCC cases (UALCAN database). **B.** Correlation of the *CDK1*, *CDK5*, *CDC20*, *CCNA2*, *CCNB1*, and *CCNB2* expression with tumor grade among HCC cases (UALCAN database). **C.** Correlation of the *CDK1*, *CDK5*, *CDC20*, *CCNA2*, *CCNB1*, and *CCNB2* expression with tumor stage among HCC cases (TISIDB database). **D.** Correlation of the *CDK1*, *CDK5*, *CDC20*, *CCNA2*, *CCNB1*, and *CCNB2* expression with tumor grade among HCC cases (TISIDB database).

Relationship between Elevated *CDK1*, *CDK5*, *CDC20*, *CCNA2*, *CCNB1*, and *CCNB2* mRNA Expression and Dismal Prognosis for HCC Cases.

The crucial cell cycle-related genes efficiency in HCC patient survival was also found. The UALCAN database was utilized to examine the relationship of mRNA expression of *CDK1*, *CDK5*, *CDC20*, *CCNA2*, *CCNB1*, and *CCNB2* with HCC patient survival based on the public datasets. Our

results suggested that increased *CDK1* (*P* = 0.0001), *CDK5* (*P* = 0.0011), *CDC20* (*P* = 0.0001), *CCNA2* (*P* = 0.0013), *CCNB1* (*P* = 0.0001), and *CCNB2* (*P* = 0.00055) showed a significant relationship with poor OS. (**Supplement Figure 3**).

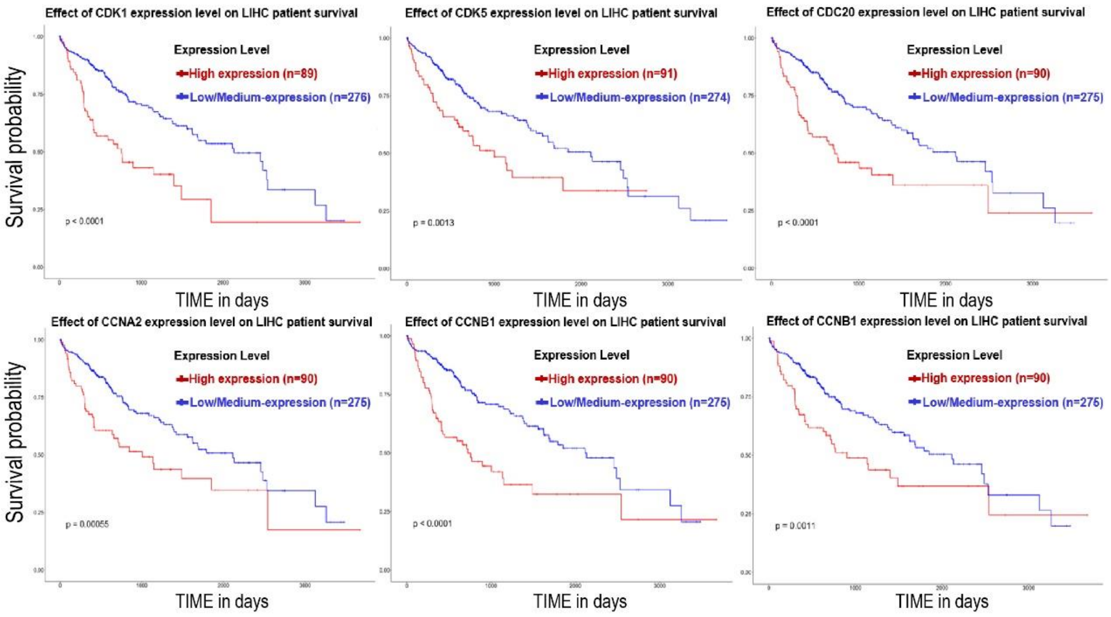

SUPPLEMENTARY FIGURE 3 | Significance of the *CDK1*, *CDK5*, *CDC20*, *CCNA2*, *CCNB1*, and *CCNB2* mRNA expression in predicting the prognosis for HCC cases (UALCAN database).

***CDK1*, *CDK5*, *CDC20*, *CCNA2*, *CCNB1*, and *CCNB2* Expression Is Correlated with Immune Infiltration Level in HCC.**

We also used TIMER database to analyze whether CDK1, CDK5, CDC20, CCNA2, CCNB1 and CCNB2 expression were correlated with immune infiltration levels in HCC. Our results showed that the levels of cell cycle-related genes expression had remarkable correlations with B cell, CD8+ T cells, CD4+ T cells, neutrophils, macrophages, and DCs in HCC **(Figure 5)**. The *P*-value were listed in **Supplementary Table 2.**

SUPPLEMENTARY TABLE 2 | *P*-value of the cell cycle-related genes expression correlations with B cell, CD8+ T cells, CD4+ T cells, neutrophils, macrophages, and DCs in HCC.

Gene	B cell	CD8+ T cell	CD4+ T cell	Macrophage	Neutrophils	Dendritic cell
CDK1	**2.97e-20**	**2.38e-09**	**2.72e-10**	**2.60e-18**	**4.98e-11**	**1.17e-17**
CDK5	**1.28e-02**	0.305	0.0704	**1.31e-02**	**6.89e-04**	**1.64e-02**
CDC20	**8.39e-19**	**2.01e-11**	**4.78e-07**	**6.63e-16**	**2.12e-09**	**3.54e-19**
CCNA2	**7.31e-21**	**1.63e-11**	**1.60e-09**	**1.21e-14**	**2.41e-12**	**8.02e-22**
CCNB1	**2.94e-20**	**1.12e-08**	**9.33e-08**	**5.42e-16**	**6.81e-11**	**1.15e-16**
CCNB2	**6.57e-22**	**2.78e-09**	**7.86e-09**	**6.27e-18**	**1.49e-09**	**6.57e-19**

Bold values indicate statistically significant ($P < 0.05$).

SUPPLEMENTARY TABLE 3 | Correlation analysis between cell cycle-related genes and markers of immune cells in TIMER.

Description	Gene markers	CDK1		CDK5		CDC20	
		Cor	*P*	Cor	*P*	Cor	*P*
**CD8+ T cell**	CD8A	0.198	***	-0.04	0.438	0.218	***
	CD8B	0.184	***	0.009	0.857	0.256	***
**T cell (general)**	CD3D	0.274	***	0.06	0.25	0.364	***
	CD3E	0.202	***	0.124	**	0.217	***
	CD2	0.216	***	0.12	*	0.243	***
**B cell**	CD19	0.273	***	0.043	0.408	0.275	***
	CD79A	0.158	***	0.18	***	0.165	*
**Monocyte**	CD86	0.284	***	0.069	***	0.326	***
	CD115 (CSF1R)	0.131	*	0.088	0.0918	0.179	***
**TAM**	CCL2	0.039	0.459	0.02	0.701	0.038	0.464
	CD68	0.23	***	0.076	0.142	0.242	***
	IL10	0.219	***	0.012	0.822	0.23	***
**M1 Macrophage**	INOS (NOS2)	0.02	0.695	0.116	*	0.098	0.0588
	IRF5	0.394	***	0.367	***	0.325	***
	COX2(PTGS2)	0.101	0.0526	-0.109	*	0.034	0.514
**M2 Macrophage**	CD163	0.067	0.0197	0.043	0.406	0.046	0.38
	VSIG4	0.08	0.123	0.149	*	0.098	0.0588
	MS4A4A	0.089	0.0859	0.056	0.285	0.089	0.0869
**Neutrophils**	CD66b (CEACAM8)	0.123	*	0.031	0.558	0.092	0.0775
	CD11b (ITGAM)	0.257	***	0.223	***	0.317	***
	CCR7	0.089	0.0873	0.195	***	0.035	0.499
**Natural killer cell**	KIR2DL1	0.035	0.5	0.042	0.418	0.058	0.262
	KIR2DL3	0.167	**	0.076	0.145	0.139	**
	KIR2DL4	0.195	***	0.134	***	0.228	***
	KIR3DL1	0.01	0.843	0.007	0.893	0.033	0.528
	KIR3DL2	0.085	0.102	-0.012	0.821	0.082	0.113
	KIR3DL3	0.064	0.219	-0.022	0.675	0.062	0.231
	KIR2DS4	0.075	0.148	0.02	0.708	0.018	0.73
**Dendritic cell**	HLA-DPB1	0.154	**	0.03	0.563	0.196	***
	HLA-DQB1	0.132	*	0.025	0.628	0.193	***
	HLA-DRA	0.17	***	0.027	0.601	0.18	***
	HLA-DPA1	0.141	**	0.041	0.426	0.151	*
	BDCA-1(CD1C)	0.121	*	-0.16	*	0.063	0.225
	BDCA-4(NRP1)	0.231	***	0.145	*	0.114	*
	CD11c (ITGAX)	0.331	***	0.045	0.384	0.318	***
**Th1**	T-bet (TBX21)	0.081	0.119	-0.095	0.0683	0.073	0.159
	STAT4	0.262	***	-0.121	0.0194	0.263	***

	STAT1	0.372	***	0.031	0.552	0.287	***
	IFN-γ (IFNG)	0.266	***	0.108	*	0.319	***
	TNF-α (TNF)	0.252	***	-0.022	***	0.265	***
**Th2**	GATA3	0.208	***	-0.111	*	0.203	***
	STAT6	0.114	*	0.108	*	-0.026	0.62
	STAT5A	0.265	***	0.208	***	0.26	***
	IL13	0.068	0.19	0.024	0.641	0.101	0.052
**Tfh**	BCL6	0.161	**	0.112	0.0306	0.075	0.15
	IL21	0.156	**	-0.002	0.962	0.136	0.0872
**Th17**	STAT3	0.114	*	0.108	*	0.025	0.627
	IL17A	0.135	**	-0.118	0.0232	0.043	0.411
**Treg**	FOXP3	0.163	**	0.065	0.211	0.097	0.0612
**Treg**	CCR8	0.392	***	0.011	0.828	0.31	***
	STAT5B	0.247	***	0.225	***	0.069	0.183
	TGFβ (TGFB1)	0.278	***	0.013	0.807	0.27	***
**T cell exhaustion**	PD-1 (PDCD1)	0.33	***	-0.046	0.377	0.37	***
	CTLA4	0.357	***	-0.016	0.765	0.396	***
	LAG3	0.294	***	0.013	0.8	0.372	***
	TIM-3 (HAVCR2)	0.296	***	0.074	0.154	0.352	***
	GZMB	0.092	0.0779	0.061	0.244	0.121	*

**Description**	**Gene markers**	**CCNA2**		**CCNB1**		**CCNB2**	
		Cor	*P*	Cor	*P*	Cor	*P*
**CD8+ T cell**	CD8A	0.236	***	0.186	***	0.227	***
	CD8B	0.206	***	0.188	***	0.23	***
**T cell (general)**	CD3D	0.271	***	0.278	***	0.303	***
	CD3E	0.228	***	0.189	***	0.222	***
	CD2	0.225	***	0.205	***	0.238	***
**B cell**	CD19	0.268	***	0.232	***	0.272	***
	CD79A	0.185	***	0.137	**	0.177	***
**Monocyte**	CD86	0.322	***	0.316	***	0.318	***
	CD115 (CSF1R)	0.172	***	0.166	**	0.156	*
**TAM**	CCL2	0.076	0.144	0.034	0.512	0.065	0.211
	CD68	0.221	***	0.272	***	0.216	***
	IL10	0.247	***	0.249	***	0.235	***
**M1 Macrophage**	INOS (NOS2)	0.022	0.674	0.028	0.597	0.014	0.786
	IRF5	0.369	***	0.377	***	0.379	***
	COX2(PTGS2)	0.125	*	0.08	0.124	0.078	0.133
**M2 Macrophage**	CD163	0.125	**	0.082	0.114	0.059	0.253
	VSIG4	0.125	*	0.106	**	0.077	0.141
	MS4A4A	0.133	*	0.106	*	0.087	0.0945

**Neutrophils**	CD66b (CEACAM8)	0.091	0.0802	0.118	*	0.1	0.0546
	CD11b (ITGAM)	0.325	***	0.304	***	0.271	***
	CCR7	0.129	*	0.046	0.382	0077	***
**Natural killer cell**	KIR2DL1	0.013	0.809	0.038	0.467	0.025	0.628
	KIR2DL3	0.179	***	0.152	**	0.16	**
	KIR2DL4	0.218	***	0.195	***	0.214	***
	KIR3DL1	0.043	0.404	-0.03	0.566	-0.001	0.985
	KIR3DL2	0.07	0.18	0.055	0.288	0.096	0.0642
	KIR3DL3	0.083	0.111	0.036	0.49	0.096	0.0642
	KIR2DS4	0.088	0.089	0.037	0.474	0.052	0.317
**Dendritic cell**	HLA-DPB1	0.2	***	0.185	***	0.194	***
	HLA-DQB1	0.168	**	0.161	**	0.184	***
	HLA-DRA	0.218	***	0.196	***	0.183	***
	HLA-DPA1	0.198	**	0.166	**	0.164	*
	BDCA-1(CD1C)	0.131	*	0.108	*	0.136	**
	BDCA-4(NRP1)	0.246	***	0.183	***	0.19	***
	CD11c (ITGAX)	0.354	***	0.334	***	0.315	***
**Th1**	T-bet (TBX21)	0.098	0.0599	0.05	0.34	0.086	0.0979
	STAT4	0.263	***	0.221	***	0.249	***
	STAT1	0.387	***	0.375	***	0.366	***
	IFN-γ (IFNG)	0.274	***	0.276	***	0.293	***
	TNF-α (TNF)	0.268	***	0.279	***	0.268	***
**Th2**	GATA3	0.227	***	0.188	***	0.222	***
	STAT6	0.099	0.0575	0.054	0.287	0.048	0.354
	STAT5A	0.276	***	0.232	***	0.254	***
	IL13	0.128	0.0133	0.101	0.0527	0.101	0.0526
**Tfh**	BCL6	0.161	*	0.1	0.0554	0.089	0.0861
	IL21	0.181	***	0.171	***	0.164	*
**Th17**	STAT3	0.175	***	0.096	0.0651	0.051	0.331
	IL17A	0.109	*	0.081	0.121	0.099	0.0576
**Treg**	FOXP3	0.213	***	0.139	0.0073	0.163	*
**Treg**	CCR8	0.429	***	0.375	***	0.363	***
	STAT5B	0.302	***	0.177	***	0.195	***
	TGFβ (TGFB1)	0.275	***	0.303	***	0.295	***
**T cell exhaustion**	PD-1 (PDCD1)	0.324	***	0.323	***	0.356	***
	CTLA4	0.335	***	0.34	***	0.351	***
	LAG3	0.293	***	0.293	***	0.362	***
	TIM-3 (HAVCR2)	0.348	***	0.33	***	0.318	***
	GZMB	0.087	0.0944	0.078	0.136	0.114	*

TAM, tumor-associated macrophage; Th, T helper cell; Tfh, Follicular helper T cell; Treg, regulatory T cell; Cor, R value of Spearman's correlation. * P <0.01; ** P <0.001; *** P <0.0001.
